# Innate Immune Responses to *Cryptococcus*

**DOI:** 10.3390/jof3030035

**Published:** 2017-07-02

**Authors:** Lena J. Heung

**Affiliations:** Infectious Diseases Service, Department of Medicine, Memorial Sloan Kettering Cancer Center, New York, NY 10065, USA; heungl@mskcc.org; Tel.: +1-212-639-7980

**Keywords:** *Cryptococcus*, innate immune response, fungal recognition, host-fungus interactions

## Abstract

*Cryptococcus* species are encapsulated fungi found in the environment that predominantly cause disease in immunocompromised hosts after inhalation into the lungs. Even with contemporary antifungal regimens, patients with cryptococcosis continue to have high morbidity and mortality rates. The development of more effective therapies may depend on our understanding of the cellular and molecular mechanisms by which the host promotes sterilizing immunity against the fungus. This review will highlight our current knowledge of how *Cryptococcus*, primarily the species *C. neoformans*, is sensed by the mammalian host and how subsequent signaling pathways direct the anti-cryptococcal response by effector cells of the innate immune system.

## 1. Introduction

The encapsulated, yeast-like fungi of the genus *Cryptococcus* are prevalent throughout the environment worldwide. The most common species that cause disease in humans are *Cryptococcus neoformans* and *Cryptococcus gattii*. These pathogens can cause a life-threatening meningoencephalitis after acquisition through the respiratory tract and subsequent dissemination to the central nervous system (CNS). While *C. gattii* can infect apparently immunocompetent hosts, *C. neoformans* is more often an opportunistic pathogen, affecting immunocompromised patients including those with HIV/AIDS, cancer and solid organ transplantation [1].

Cryptococcal meningitis has been estimated to affect up to 1 million people worldwide each year [2,3]. Despite modern-day combination antifungal therapy, the mortality rate for cryptococcal meningitis is estimated at 15–25% [4,5], and the at-risk population is expanding with the development of new immunosuppressive regimens for autoimmunity and cancer [6]. More effective approaches to treating cryptococcosis may necessitate the incorporation of immunomodulatory therapies. Therefore, it is essential to understand the cellular and molecular mechanisms of immunity to *Cryptococcus* in mammalian hosts. While the adaptive immune response to *Cryptococcus* is an important arm of anti-cryptococcal immunity (reviewed in [1,7,8]), this review will focus on our current knowledge of innate immune responses to the species *C. neoformans* and identify significant questions that remain to be investigated.

## 2. Animal Models of Cryptococcosis

Different vertebrate and invertebrate animal models have been utilized in the study of cryptococcosis (for more comprehensive reviews see [1,9,10]). Predominantly, murine models have been used to study innate immune responses to *C. neoformans* due to the relative ease of genetic modification, manipulation and maintenance of this mammalian host. Therefore, results from mouse studies will comprise the majority of this review.

The use of mouse models of cryptococcosis does have its challenges. Different mouse strains develop different T helper cell (Th) responses to *C. neoformans*; mice that develop Th type 2 (Th2) responses are more susceptible to cryptococcosis, while those that develop Th type 1 (Th1) responses are more resistant [11,12,13,14,15]. Mouse susceptibility can further vary depending on the virulence of the *C. neoformans* strain, the type and amount of infectious propagule (i.e., spore versus yeast form), and the route of administration [11,12,13,14,16,17,18]. *C. neoformans* has two main variants: var. *grubii* (Serotype A), which is the most common clinical isolate, and var. *neoformans* (Serotype D) [1]. The most physiologic route of infection is through the respiratory tract, either intranasal or intratracheal. However, respiratory infection in mice can result in variable dissemination to the CNS, so systemic infection (intravenous or intraperitoneal) and direct inoculation into the cerebrospinal fluid have been used to study the pathology of *C. neoformans* in the CNS [13,19,20].

As an example of the differences between mouse models of cryptococcosis, respiratory infection of C57BL/6 mice with the highly virulent serotype A strain H99 leads to a Th2-skewed immune response that results in an acute and uniformly fatal infection [21,22,23]. On the other hand, respiratory infection of BALB/c mice with a less virulent serotype D strain like 52D leads to a Th1-skewed immune response that results in a more chronic infection that can eventually be cleared in a CD4^+^ T-cell-dependent manner [11,12,15,24]. A protective model of pulmonary cryptococcosis has also been established in which mice are infected with a *C. neoformans* strain H99-γ, that has been modified to express murine interferon gamma (IFNγ) [25].

## 3. Host Recognition of *Cryptococcus*

Fungal pathogens are typically sensed through the detection of fungal antigens, or pathogen-associated molecular patterns (PAMPs), by pattern recognition receptors (PRRs) on host immune cells. Engagement of PRRs induces signal transduction that coordinates innate immune processes like phagocytosis and cytokine production. Common fungal PAMPs include components of the cell wall, such as β-glucans, mannans, and chitin. However, *C. neoformans* provides an interesting challenge due to its polysaccharide capsule that can mask these potential PAMPs. Correspondingly, many PRRs that are known to detect other fungal pathogens, including members of the C-type lectin receptor (CLR) and Toll-like receptor (TLR) families, do not have similar roles in the recognition of *C. neoformans.* Therefore, the mechanisms by which *C. neoformans* is sensed by the host are still not fully defined.

### 3.1. C-Type Lectin Receptors

The CLRs are a large family of receptors that can recognize fungal carbohydrate ligands like β-glucans or mannans. An engaged CLR typically initiates downstream signaling pathways either through its own intracellular signaling domain, if present, or else through signaling adapters that contain an immunoreceptor tyrosine-based activation motif (ITAM), such as Fc receptor γ-chain (FcRγ or FcεRIγ chain) or DNAX activation protein of 12 kDa (DAP12). While CLRs have established roles in host innate immune responses to other pathogenic fungi (reviewed in [26]), their ability to mediate immunity to *C. neoformans* is less robust. There is evidence that β-glucans can be accessible on encapsulated yeast [27] and spore [28] forms of *C. neoformans* and that the fungal cell wall can be exposed at daughter bud sites prior to capsule assembly [29], but it is likely that the capsule is interfering with many of these potential interactions in vivo [30,31].

Mannose receptor (MR/CD206) binds to fucose and terminal mannose moieties and is known to have roles in phagocytosis as well as antigen processing and presentation as a receptor of the endocytic pathway (reviewed in [32]). MR does not have any known intracellular signaling motifs and can also exist in a soluble form [32,33], suggesting it may work in concert with other receptors for signal transduction, such as TLR2 [34]. Human MR has been shown to bind cryptococcal mannoproteins in vitro [35]. It is unclear if MR binds whole cryptococcal cells since MR-deficient murine phagocytes had no changes in binding and uptake of spores or yeast cells compared to wild-type (WT) phagocytes by microscopy [36]. Nevertheless, MR^−/−^ mice challenged with *C. neoformans* in an acute respiratory infection model appear to have a moderate increase in fungal burden and susceptibility to infection [37]. Several studies have investigated the ability of MR to facilitate the priming of adaptive T cell responses by dendritic cells (DCs). MR-deficient bone marrow-derived DCs (BMDCs) from mice had no changes in uptake of cryptococcal mannoproteins and no differences in expression of maturation markers like MHCII, CD40 and CD86 [37]. In contrast, studies with human cells indicate that blocking MR can inhibit maturation marker expression by DCs in response to cryptococcal mannoproteins [38] and can inhibit fungal uptake by DCs and subsequent lymphocyte proliferation [39]. Therefore, the mechanisms by which MR mediates innate immune responses to *C. neoformans* warrants continued investigation.

Dendritic cell-specific intercellular adhesion molecule-3 grabbing non-integrin (DC-SIGN/CD209) binds fucose and mannose residues and is involved in antigen uptake as well as cellular adhesion (reviewed in [40]). Complicating its study, DC-SIGN has eight homologs in mice, designated DC-SIGN-related proteins (SIGNR) 1-8. SIGNR3 (CD209d) is considered the closest homolog to human DC-SIGN [41], and SIGNR3 and SIGNR1 (CD209b) are the only homologs shown to bind fungal ligands [42]. Human DC-SIGN binds cryptococcal mannoproteins in vitro [43], but murine SIGNR1 does not influence the ability of splenic macrophages to internalize the cryptococcal capsular polysaccharide glucuronoxylomannan (GXM) [44]. Additional studies on the potential role of DC-SIGN or its murine homologs during cryptococcal infection are currently lacking.

Collectins are secreted carbohydrate-binding proteins and include the lung surfactant proteins (SPs) SP-A and SP-D and serum mannose binding lectin (MBL), also referred to as mannose binding protein (MBP). Collectins have been shown to engage with various fungal pathogens [45,46,47,48] and regulate cytokine responses by binding to cell surface receptors like CD14, TLR2 and TLR4 [49,50]. Interestingly, SP-D appears to be detrimental to the host, as SP-D^−/−^ mice have improved survival after infection with *C. neoformans* [51]. SP-D binds to and protects *C. neoformans* from macrophage killing, and its activity has been correlated with increased IL-5 production and pulmonary eosinophilia [29,52]. The cryptococcal PAMP recognized by SP-D in vivo is unclear. In vitro, SP-D can bind to capsular GXM and mannoprotein 1 (MP1), but has higher affinity to pustulan, an analog of β-1,6-glucan found in the cryptococcal cell wall [29]. This higher affinity for a cell wall component correlates with the observation that acapsular *C. neoformans* mutants are more susceptible to agglutination and phagocytosis in the presence of SP-D compared to encapsulated strains [29,53,54]. Further studies are needed to determine which interactions and signaling mechanisms are essential for the harmful effects of SP-D on the host response. In contrast, SP-A can bind to *C. neoformans* but does not affect phagocytosis [55] and does not regulate murine susceptibility to infection [56]. MBL is known to bind mannose and *N*-acetylglucosamine (GlcNAc) and has been shown to act as an opsonin for complement activation [57]. However, soluble human MBL can only bind acapsular *C. neoformans* and minimally improves phagocytosis of these fungal cells by human polymorphonuclear cells in vitro [30,54,58,59]. Thus, the overall role of collectins in anti-cryptococcal responses appears to be minimal or else harmful to the host.

Other CLRs have been investigated but do not appear to have links to anti-cryptococcal immunity. Dectin-1 (CLEC7A) does not mediate immune responses in vitro or in vivo to either yeast or spore forms of *C. neoformans* [36,60]. Co-expression of Dectin-1 and TLR2 in vitro also does not facilitate signal transduction in response to the fungus [61]. Dectin-2 (CLEC6A/CLEC4N) is not essential in host defense against *C. neoformans* yeast or spore forms despite molecular evidence of increased Th2 and decreased Th1 responses in Dectin-2^−/−^ mice [36,62]. Dectin-3 or macrophage C-type lectin (MCL/CLEC4D/CLECSF8) does not regulate murine outcomes after *C. neoformans* infection or phagocytosis of fungal cells [63,64] and cannot initiate signal transduction in response to *C. neoformans* spores [36]. Macrophage inducible C-type lectin (Mincle) does not bind *C. neoformans* or induce signal transduction in response to the fungus in vitro [36]. Langerin (CD207) does not bind to either encapsulated or acapsular *C. neoformans* [65]. Work remains to determine whether other CLRs, including novel receptors like CD23/FcεRII [66], may play a role in host recognition of *C. neoformans*.

### 3.2. Toll-Like Receptors

The potential role of TLRs as cryptococcal PRRs has been supported by evidence that myeloid differentiation primary response gene 88 (MyD88), a signaling molecule downstream of most TLRs, plays a role in murine anti-cryptococcal responses [67,68,69]. However, direct experimental evidence supporting a role for many of the TLRs in cryptococcosis is limited. Whether TLR signaling is relevant to human disease is unclear, as people with Mendelian defects in MyD88 do not have increased susceptibility to cryptococcosis [70,71].

Studies on TLR2 have had conflicting results regarding the ability of this receptor to influence infectious outcomes and to initiate signal transduction in response to *C. neoformans*, perhaps related to differences in experimental design. Biondo et al. demonstrated that TLR2^−/−^ mice have increased susceptibility to systemic (intraperitoneal) infection with *C. neoformans,* as measured by survival, organ fungal burden and cytokine production [67]. Yauch et al. found that TLR2^−/−^ mice have increased susceptibility to respiratory infection but not systemic (intravenous) infection; however, there were no differences in lung fungal burden or cytokine production in the TLR2^−/−^ mice compared to WT mice [68]. Nakamura et al. also found no differences in fungal burden or cytokine production in TLR2^−/−^ mice infected through the respiratory tract, and *C. neoformans* did not induce nuclear factor kappa-light-chain-enhancer of activated B cells (NF-κB) activation through TLR2 in an in vitro cell reporter assay, even with co-expression of Dectin-1 [61].

TLR4, in conjunction with its co-receptor CD14, can respond to cryptococcal GXM in vitro by inducing NF-κB but not mitogen activated protein (MAP) kinase pathways or tumor necrosis factor alpha (TNFα) secretion, suggesting incomplete activation [72]. Monoclonal antibodies against TLR4 can inhibit Fas ligand expression [73] and partially block GXM uptake by human peripheral blood mononuclear cell (PBMC)-derived macrophages [74]. However, TLR4 has not been shown to regulate murine susceptibility to infection [67,68].

The strongest evidence for direct TLR involvement in anti-cryptococcal responses is for TLR9, an intracellular receptor of the endocytic pathway that typically recognizes unmethylated cytosine-phosphate-guanine (CpG) motifs common in the DNA of bacteria and viruses (reviewed in [75,76]). More recently, the fungus *Aspergillus fumigatus* was found to contain unmethylated CpG motifs that can stimulate cytokine responses by DCs in vitro in a TLR9-dependent manner [77]. Several groups have also used synthetic CpG-oligodeoxynucleotides to boost the immune response against *C. neoformans* [78,79,80,81]. TLR9^−/−^ mice are more susceptible to cryptococcosis, potentially due to decreased recruitment and maturation of DCs and the development of Th2 immune responses, including alternative activation of macrophages [69,82,83,84]. Cryptococcal DNA can stimulate in vitro cytokine responses by DCs, which can be partially inhibited by deletion of TLR9 or MyD88 [84]. Subsequently, it has been shown that polymerase chain reaction (PCR) products amplified from cryptococcal genes involved in virulence including *URA5*, *CNLAC1*, and *CAP59* can induce the same cytokine responses by DCs [85]. Interestingly, these genes do not contain canonical CpG motifs. Thus, cryptococcal DNA can function as a PAMP for TLR9, but the specific nucleic acid motifs involved in its recognition have not been elucidated.

### 3.3. Nucleotide-Binding Oligomerization Domain (NOD)-Like Receptors

The NOD-like receptors, or nucleotide-binding domain leucine-rich repeat-containing receptors (NLRs), are a family of cytoplasmic receptors that, upon activation, can form an inflammasome complex that cleaves and activates pro-IL-1β and pro-IL-18 generated after initial microbial detection induces NF-κB (reviewed in [86]). NLR family, pyrin domain-containing 3 (NLRP3) is an NLR that has been shown to play a role in immunity against *A. fumigatus* and the yeast *Candida albicans* [87,88,89], although the ligand for NLRP3 remains unidentified. Biofilms of encapsulated *C. neoformans*, opsonized and encapsulated *C. neoformans*, and acapsular yeast forms of *C. neoformans* stimulate formation of the NLRP3 inflammasome, and mice deficient in components of the NLRP3 inflammasome are more susceptible to infection [90,91,92]. However, additional studies will be needed to further clarify the role of NLRs and inflammasome formation in antifungal responses to *C. neoformans*.

### 3.4. Scavenger Receptors

Scavenger receptors are classically known to bind and internalize oxidized low-density lipoproteins. In more recent years, they have been found to have very diverse ligands and can serve as PRRs that detect microbial PAMPs and complex with other receptors like TLRs (reviewed in [93]). In vitro studies indicate that the scavenger receptors CD36 and scavenger receptor class F member 1 (SCARF1), also known as scavenger receptor expressed by endothelial cells 1 (SREC1), can bind to and internalize encapsulated *C. neoformans*, thereby inducing cytokine responses that can further be enhanced by synergy with TLR2; competition assays suggest that CD36 and SCARF1 may bind to β-glucans and, to a lesser extent, mannan, although they do not contain classic lectin-binding domains [94]. In the same study, neutralizing anti-SCARF1 antibody inhibited binding of *C. neoformans* to alveolar macrophages in vivo, CD36^−/−^ mice were found to be more susceptible to systemic infection with *C. neoformans*, and deletion of CD36 and SCARF1 orthologues in the nematode *Caenorhabditis elegans* resulted in increased susceptibility to fungal challenge.

Macrophage receptor with collagenous structure (MARCO) has been shown to enhance early lung recruitment of monocyte-derived immune cells and protective cytokine responses after murine respiratory infection with *C. neoformans* that correlate with a transient improvement in fungal clearance [95]. Interestingly, MARCO-deficient macrophages and DCs exhibit no defect in fungicidal activity though they do have decreased interactions with fungal cells [95].

Scavenger receptor A (SRA/SR-AI/II/CD204/SCARA1) has been reported to have detrimental effects on host immunity to *C. neoformans*. SRA^−/−^ mice have decreases in lung fungal burden likely related to regulation of cytokine responses that influence innate immune cell recruitment and activation [96]. The potential cryptococcal ligands for SRA and MARCO and additional mechanistic details for how all these scavenger receptors influence anti-cryptococcal responses have not yet been determined.

### 3.5. Natural Antibodies

Natural antibodies, that are predominantly of the immunoglobulin M (IgM) isotype, are constitutively produced in mammalian hosts by an innate subset of B lymphocytes called B-1 cells; opsonization of microbial antigens with natural IgM can result in complement activation, phagocytosis by macrophages, and priming of adaptive immune responses (reviewed in [97,98]). It has been shown that IgM produced by murine B-1 cells in vitro can bind to cell wall laminarin, capsular GXM, and acapsular and heat-killed encapsulated *C. neoformans* [99]. Secretory IgM-deficient (sIgM^−/−^) mice have increased susceptibility to respiratory infection with *C. neoformans* compared to control mice and exhibit defects in Th1 polarization and phagocytosis of fungi by alveolar macrophages; the defect in phagocytosis can be ameliorated by administration of IgM into the lungs [100]. Additionally, depletion of B-1 cells in pulmonary infected mice increases fungal burden and decreases phagocytosis of fungal cells by alveolar macrophages compared to non-depleted controls; adoptive transfer of B-1 cells into depleted mice can restore the phenotype to that of control mice [99]. On the other hand, sIgM^−/−^ mice infected systemically with *C. neoformans* have improved survival compared to control mice [101]. It was found that these sIgM^−/−^ mice have an increased baseline number of B-1 cells [101], and, interestingly, B-1 cell derivatives may have direct fungicidal effects against *C. neoformans* [102]. Thus, IgM and B-1 cells may play different roles in the anti-cryptococcal response depending on the tissue compartment. X-linked immunodeficient (XID) mice, that have a defect in B cell development and IgM production due to a mutation in Bruton’s tyrosine kinase (Btk), exhibit increased susceptibility to both respiratory and systemic infection with *C. neoformans* [6,103]. However, adoptive transfer of B-1 cells into pulmonary infected XID mice could neither reverse this susceptibility to *C. neoformans* nor fully restore serum IgM levels, suggesting that B-1 cells may not be the only source of protective IgM or that additional immune mechanisms are contributing to the phenotype in this particular model [6].

Human studies support a role for IgM in protective immune responses against *C. neoformans.* The percentage of IgM-expressing memory B cells inversely correlates with the risk for developing cryptococcosis among HIV-positive patients [104]. In solid organ transplant recipients, pre-transplantation levels of GXM-reactive IgM inversely correlate with the development of post-transplant cryptococcosis [105]. The ability to identify B-1 cells in humans has recently been reported (reviewed in [106]), which may facilitate future studies on the role of these innate immune cells and natural antibodies in human cryptococcosis.

### 3.6. Complement and Other Soluble Mediators

The complement system is an important mediator for the phagocytosis of *C. neoformans* by innate immune cells. Opsonization by complement has been shown to improve uptake and killing of *C. neoformans* by phagocytes [107,108] and to mediate DC responses to *C. neoformans* [109]. Activation of complement can occur through three pathways: alternative, classical, and lectin (reviewed in [110]). Disruption of the alternative, but not the classical, pathway of complement reduces phagocytosis of *C. neoformans* in vitro and increases the mortality of guinea pigs after infection [111,112]. The lectin pathway likely does not play a significant role given minimal interactions between MBL and *C. neoformans*, as discussed earlier in this review. It has been shown that complement component 3 (C3) binds to the capsule of *C. neoformans* and then is degraded to inactivated C3b (iC3b) [113,114,115]. Phagocytosis can then proceed via the action of complement receptors (CR). Blocking CR1, CR3 and CR4 decreases the interaction between *C. neoformans* and human macrophages in vitro [108]. CR3 has been shown to facilitate complement-mediated phagocytosis of *C. neoformans* by murine macrophages [116], but CR3 and CR4 can also mediate phagocytosis independent of complement [117]. Additionally, signaling by C5a through its receptor C5aR appears to be important for neutrophil uptake and killing of *C. neoformans* in mice [118].

Other potential soluble mediators of anti-cryptococcal immunity have been studied. Pentraxin 3 (PTX3) expression is induced in the brains of mice infected intracerebrally with *C. neoformans* [119], but it is not yet known what function PTX3 may play in anti-cryptococcal responses. Recombinant rat ficolin-A can bind and facilitate uptake of acapsular mutants of *C. neoformans* by lung epithelial cells in vitro but does not bind encapsulated *C. neoformans* [120], so it is unclear if ficolins play any significant role in anti-cryptococcal immunity. Finally, production of antimicrobial peptides is increased in a protective model of cryptococcosis [121], but their specific functions in the response to *C. neoformans* are not understood.

### 3.7. Other Recognition Pathways

Additional potential cryptococcal PAMPs have been identified, but their receptors remain unclear. Chitin is a long chain polymer of GlcNAc that can also be deacetylated to chitosan; both forms are components of the cryptococcal cell wall [1] and appear to have detrimental effects on the host immune response upon recognition. The chitin content of cryptococcal cells has been shown to correlate with Th2 cell accumulation and increased mortality in the murine host [122], and a chitosan-deficient strain of *C. neoformans* promotes protective Th1 host responses and is avirulent in mice [123]. Cryptococcal chitin has been shown to induce IL-10 secretion from human and murine macrophages [124] and induce Th2 responses through CD11b^+^ conventional DCs, although this process does not seem to occur through direct sensing of chitin by DCs [122]. The PRRs for chitin and chitosan are still unknown (reviewed in [125]). Studies using *C. albicans*-derived chitin suggest that chitin recognition is dependent on MR, NOD2, and TLR9 [124], and purified chitosan can induce inflammasome activation [126,127]. The hypervirulent *rim101*Δ *C. neoformans* mutant, that has increased chitosan content and exposure of chito-oligomers on its cell surface, induces TNFα secretion by murine bone marrow-derived macrophages but not IL-1β, suggesting cryptococcal chitosan does not induce the inflammasome; however, the induction of TNFα appears to be dependent on the caspase recruitment domain-containing 9 (CARD9) and MyD88 signaling molecules, indicating a potential role for CLRs and TLRs [128,129,130].

Another possible source of cryptococcal PAMPs are extracellular vesicles (EVs), also referred to as exosomes, which are bilayer vesicles released by *C. neoformans* [131] and can contain an array of cellular components including polysaccharides, nucleic acids, and proteins (reviewed in [132,133]). Although some EVs may be able to promote the virulence of *C. neoformans* [134,135], cryptococcal EVs have also been shown to be internalized by macrophages and stimulate cytokine secretion, NO production, and uptake and killing of the fungus in vitro [136]. As we improve our technical capability to isolate extracellular vesicles, it will be interesting to perform further analysis of their contents under different host conditions and determine if there are specific EV-borne PAMP interactions with host PRRs.

## 4. Intracellular Signaling Molecules

Another approach to defining innate immune responses to *C. neoformans* has been to study the role of molecules that commonly integrate signals from PRRs after fungal recognition. These include CARD9, MyD88, and the signaling adapters DAP12 and FcRγ (reviewed in [137,138]).

CARD9 is best known as a downstream mediator of signaling through CLRs like Dectin-1, but it can also transmit signals from TLRs and NOD2 and can facilitate activation of NF-κB or MAP kinase pathways (reviewed in [139,140]). Respiratory infection of CARD9^−/−^ mice with *C. neoformans* results in increased lung fungal burden and neutrophilia along with defective early IFNγ production by NK cells and memory T cells [141]. CARD9 may not play a direct role in phagocytic pathways, as CARD9-deficient murine phagocytes have no defect in binding or uptake of *C. neoformans* spores or yeast forms as evaluated by microscopy [36], but it may regulate cytokine responses. For example, TNFα production is reduced in CARD9-deficient murine macrophages in response to the chitosan-enriched, acapsular *rim101*Δ *cap59*Δ *C. neoformans* mutant [128]. Together, these studies suggest that CARD9 may play a role in the host response to *Cryptococcus*, but the signaling pathway requires further definition.

MyD88 has well-established roles in signal transduction for most TLRs but can also function downstream of the cytokine receptors IL-1R and IL-18R [142,143]. MyD88^−/−^ mice have increased susceptibility to both systemic and respiratory infection with *C. neoformans* [67,68,69]. Since the increased susceptibility of TLR2^−/−^ mice to cryptococcosis is not as pronounced as that of MyD88^−/−^ mice [67,68], MyD88 may mediate non-TLR signaling in response to *C. neoformans* as well. Indeed, IL-18R^−/−^ mice, but not IL-1R^−/−^ mice, have increased susceptibility to respiratory infection with *C. neoformans*, and knockout of either receptor causes significant changes in lung cytokine production compared to WT mice [69]. Additionally, mice deficient in IL-18 have increased susceptibility to cryptococcosis [144,145]. Thus, MyD88 may integrate signals from multiple cryptococcal recognition pathways during the host innate immune response.

DAP12 is an ITAM-containing signaling adapter that pairs to a variety of carbohydrate- and protein-binding immunoreceptors on myeloid and NK cells, including CLRs and other tyrosine kinase-signaling receptors (reviewed in [138,146,147,148]). DAP12 has been shown to have roles in the regulation of macrophage activation and survival [149,150]. Interestingly, DAP12-deficient macrophages have enhanced fungal uptake and killing and TNFα production in response to *C. neoformans*, and DAP12^−/−^ mice are more resistant to respiratory infection with *C. neoformans* than WT mice [21]. Thus, DAP12 appears to inhibit beneficial fungicidal macrophage responses to *C. neoformans*. Further research will be needed to identify the DAP12-associated PRRs that trigger these immunosuppressive effects and could be potential immunomodulatory targets for the treatment of cryptococcosis.

FcRγ is also an ITAM-containing signaling adapter utilized by receptors on myeloid and NK cells (reviewed in [138]). In contrast to DAP12, there is no current evidence that supports a role for FcRγ in innate immune responses to *C. neoformans*. Murine phagocytes from FcRγ^−/−^ mice demonstrate no changes in binding or uptake of spores or yeast [36]. Any other potential roles of FcRγ during cryptococcosis are still unknown.

Additional important signaling molecules in fungal sensing pathways, including spleen tyrosine kinase (Syk), have not yet been investigated for their roles in cryptococcosis. As these gaps in our knowledge are filled, we may gain further insight into the signaling network that enables coordination of the innate immune response by effector cells.

## 5. Effector Functions of Innate Immune Cells

After a fungal pathogen is recognized by the innate immune system, signal transduction coordinates the effector functions of innate immune cells, which may include phagocytosis and the generation of inflammatory response mediators such as cytokines, fungicidal compounds and acute phase reactants. These processes can regulate clearance of the fungus or initiate the development of adaptive immune responses. In the case of *C. neoformans*, these pathways can also be subverted by the pathogen to suppress the host innate immune response and allow the fungus to proliferate instead.

### 5.1. Inflammatory Monocytes

Inflammatory monocytes are innate immune cells that are recruited from the bone marrow to sites of infection or inflammation, whereupon they can differentiate into macrophages or DCs [151,152,153]. Although monocytes from HIV-positive patients have been reported to have impaired chemotaxis and cytotoxicity [154,155], studies using human monocytes and macrophages have had conflicting results about the role of these cells during cryptococcosis. Some researchers have found that human PBMCs can kill *C. neoformans* in vitro [156,157,158], and blood monocyte deactivation was associated with early mortality in HIV-associated cryptococcal meningitis [159]. In other studies, human PBMCs and monocyte-derived macrophages were merely fungistatic [160] or even permissive for intracellular cryptococcal proliferation and dissemination [161,162,163], and there was no difference in antifungal activity of monocyte-derived macrophages from cryptococcosis patients compared to normal controls [161].

In mice, inflammatory monocytes are defined as cells expressing lymphocyte antigen 6 complex, locus C1 (Ly6C) and C-C chemokine receptor type 2 (CCR2) that can migrate in response to the chemokines monocyte chemoattractant protein (MCP1), also known as C-C chemokine ligand 2 (CCL2), and CCL7 (reviewed in [152]). In chronic models of respiratory cryptococcosis, inflammatory monocytes appear to be beneficial to the host because CCR2^−/−^ mice, that have a defect in monocyte recruitment, develop Th2 responses and have increased fungal burden and decreased lung macrophages, CD11b^+^ DCs and CD8^+^ T cells [164,165,166]. Further, in response to infection with *C. neoformans*, Ly6C^hi^ CCR2^+^ monocytes differentiate into fungicidal exudative macrophages and CD11b^+^ DCs that promote fungal clearance and Th1 adaptive immune responses, respectively [166,167]. However, it is interesting to note that in an acute model of respiratory cryptococcosis, enhancing Th2 responses worsens survival and correlates with increased recruitment of monocytes to the lungs [122], suggesting that monocytes and their derivatives could play different roles depending on the host environment. This theory could potentially account for the differences observed in studies on human monocyte responses to *C. neoformans*.

### 5.2. Macrophages

Macrophages are phagocytic cells that include tissue-resident, embryonic-derived cells like lung alveolar macrophages as well as monocyte-derived macrophages that are of hematopoietic cell origin [168]. Since macrophages, in the guise of alveolar macrophages, are present in the lung at the time that *C. neoformans* is inhaled into the lungs, they have long been considered to be the first line innate immune cell in host defense against the fungus. Indeed, fungi are seen within lung macrophages in patients with cryptococcosis [169], and in murine models, alveolar macrophages have been visualized to quickly take up cryptococcal cells after respiratory infection [170,171]. However, there have been differing results regarding the ability of macrophages to clear *C. neoformans* from the host. While some groups have observed that murine macrophages can kill *C. neoformans* in vitro [172,173], others have found that the fungus can actually replicate within these cells, which may lead to dissemination by way of a Trojan Horse mechanism [170,174,175,176]. Interestingly, clinical *C. neoformans* isolates that exhibit higher rates of uptake by macrophages in vitro predict poor patient outcomes [177]. In murine respiratory infection models, depletion of macrophages using liposomal clodronate reduces fungal burden [176,178]. In contrast, ablation of macrophages, along with DCs, using transgenic CD11c-diphtheria toxin receptor (DTR) mice was found to worsen survival without any differences in lung fungal burden [179]. It is important to note that the ablation protocol for CD11c-DTR mice can induce fatal toxicity, even in the absence of any infection (reviewed in [180]). Thus, it will be necessary to confirm this result using alternative strategies.

It has become apparent that macrophage polarization may be a key determinant of whether macrophages are beneficial or detrimental during cryptococcosis. M1 (classically activated) macrophages produce nitric oxide (NO) through inducible NO synthase (iNOS) expression, secrete TNFα, and are fungicidal against *C. neoformans*, while M2 (alternatively activated) macrophages typically express the markers arginase 1 (Arg1), chitinase-like 4 (Chil4 or Ym2), resistin like alpha (Retnla or Fizz1), and MR (CD206) and are permissive for fungal growth (reviewed in [181]). M2 polarization has been associated with severe cryptococcal disease in non-HIV patients [182], though not in HIV-positive patients [159]. In mice, alternative activation of macrophages worsens cryptococcosis in the brain [183]. In a chronic respiratory infection model in mice, lung macrophages cycle from a resting state to an M2 phenotype, that corresponds with initial proliferation of *C. neoformans* in the lungs, followed by an M1 phenotype, that correlates to a period of fungal clearance, and then back to a resting state; this cycling could be simulated in vitro by modifying the cytokine environment with either IFNγ (M1) or IL-4 (M2) [184,185]. IFNγ^−/−^ mice have increased lung fungal burden and demonstrate alternative activation of macrophages after pulmonary challenge with *C. neoformans* [185,186]. IL4^−/−^ mice have improved fungal clearance and demonstrate classical activation of macrophages [185,187]. *C. neoformans* cells weakly stimulate expression of iNOS and Arg1 in murine macrophages in vitro, suggesting that direct interaction between fungus and phagocyte is not the only determinant of macrophage polarization [184].

From a therapeutic perspective, it will be helpful to further dissect the signaling mechanisms that can influence the polarization of macrophages during cryptococcosis. Various signaling components have been identified, including DAP12 [21], heat shock protein 70 (Hsp70) [188], and signal transducer and activator of transcription 1 (STAT1) [189,190]. Studies on other intracellular pathogens suggest that TLR signaling can induce Arg1 in macrophages [191]. Understanding these processes will allow testing of the idea that macrophage polarization drives infectious outcomes in mammalian hosts and could lay the foundation for potential new immunomodulatory strategies for the treatment of cryptococcosis.

### 5.3. Dendritic Cells

The primary function of DCs in antifungal responses is to take up, process and present antigens to prime T cells and trigger adaptive immunity (reviewed in [192,193,194]). DCs are a heterogeneous group of cells whose classification continues to evolve. Generally, it is recognized that the main subsets of DCs include classical or conventional DCs (cDCs), monocyte-derived DCs (moDCs), plasmacytoid DCs (pDCs), and Langerhans cells (reviewed in [151,195]).

DCs appear to have roles in protective immunity against *C. neoformans*. Ablation of DCs, along with macrophages, using CD11c-DTR mice increases murine mortality after infection [179], although there are limitations to this mouse model as mentioned previously in this review. DCs have been shown to take up and present cryptococcal glycoantigens [43]. Researchers have found that protective adaptive immune responses to cryptococcal antigen can be mediated by CD11b^+^ DCs and Langerhans cells [196], and moDCs have been shown to enhance Th1 responses after respiratory infection with *C. neoformans* [166]. Cryptococcal cells and cryptococcal antigen have been shown to stimulate IL-12 and IL-23p40 secretion and expression of activation markers by DCs in vitro [38,197]. DCs upregulate the CD80 activation marker in response to pulmonary *C. neoformans* challenge in vivo and can stimulate T cell activation ex vivo [198]. In addition, DCs can phagocytose and kill *C. neoformans* [39,109,198]. However, CD11b^+^ cDCs can also mediate harmful Th2 immune responses stimulated by chitin, as discussed earlier in this review [122].

The potential role of pDCs during cryptococcosis has not been as closely examined as that of cDCs and moDCs. *C. neoformans* does not appear to activate pDCs in vitro [197]. Other reports suggest that pDCs phagocytose *C. neoformans* and limit fungal growth through a Dectin-3 and ROS-dependent mechanism [63]. However, infectious outcomes are not altered in Dectin-3^−/−^ mice [63,64].

### 5.4. Neutrophils

Neutrophils are granulocytes that can phagocytose microorganisms, release antimicrobial enzymes, and produce neutrophil extracellular traps (NETs) (reviewed in [137]). Neutrophils have established roles in the innate immune response to fungal pathogens like *A. fumigatus* [199], but their role in anti-cryptococcal immunity remains poorly defined. Human neutrophils can kill *C. neoformans* in vitro [157,200], and treatment of mice with human recombinant granulocyte-colony stimulating factor (G-CSF) in combination with fluconazole improves survival from intracerebral infection [201]. At the same time, *C. neoformans* can inhibit human neutrophil migration [202], and its capsule blocks neutrophil binding of fungal cells [203]. Human neutrophils release NETs in response to acapsular *C. neoformans* mutants and the capsular polysaccharide glucoronoxylomannogalactan (GXMGal) but not in response to encapsulated *C. neoformans* or capsular GXM [204]. However, if already formed, NETs can kill encapsulated *C. neoformans* [204].

In a systemic model of murine cryptococcosis, anti-Ly6G (1A8) antibody depletion of neutrophils suggests that these cells are needed for fungal clearance in the brain and lungs [205], and neutrophils have been visualized to swarm the fungus for removal from the brain microvasculature [206,207]. In a protective model of cryptococcosis, neutrophils are the primary source of IL-17A that enhances protective immune responses, although they are not essential as γδ T cells can produce IL-17A in their absence [208]. On the other hand, after pulmonary challenge with *C. neoformans*, depletion of neutrophils and inflammatory monocytes with anti-Gr-1 (RB6-8C5) antibody improves murine survival and causes an overall reduction in inflammatory lung damage, suggesting a detrimental role for neutrophils [209]. In the same study, treatment with anti-Gr-1 had no effect on murine survival after systemic infection. Further supporting a harmful role for neutrophils, mice with genetically-induced neutrophilia appear to have increased susceptibility to cryptococcal disease [210]. Therefore, the role of neutrophils in anti-cryptococcal responses is still not clear and may depend on the specific host and/or tissue environment.

### 5.5. Natural Killer Cells

NK cells are cytotoxic lymphocytes of the innate immune system. Studies in murine models of systemic cryptococcosis suggest that NK cells may participate in early anti-cryptococcal immune responses through direct fungal interactions [211,212,213,214,215,216]. Other groups find that instead of direct cytotoxic effects against *C. neoformans*, NK cells may enhance the fungicidal activity of macrophages in mice by producing IFNγ [217,218]. Mice lacking NK cells have increased fungal burden, but they do not have increased susceptibility to infection [211,213].

The role of NK cells in anti-cryptococcal responses has been more closely examined in human cells. NK cells from HIV-positive patients are impaired in their growth inhibition of *C. neoformans* [219]. Human lymphocytes and NK cells have been shown to inhibit cryptococcal growth through direct interaction [220,221]. In studies using human primary NK cells or cell lines, Mody and colleagues have demonstrated that binding of *C. neoformans* by NK cells leads to signaling through the PI3K-ERK1/2 pathway [222] and triggers perforin degranulation to facilitate cryptococcal killing [223]. The natural cytotoxicity receptor NKp30, an immunoglobulin-like protein, has been identified as a human NK cell PRR for *C. neoformans* [224]. In the same study, blocking NKp30 impaired PI3K-ERK1/2 signaling, perforin release and ultimately fungal killing in response to *C. neoformans*. Additionally, it was shown that NK cells from HIV patients have decreased expression of NKp30 and decreased toxicity against *C. neoformans*, both of which can be reversed by IL-12 treatment in vitro. Work remains to identify any additional cryptococcal PRRs on NK cells as well as the cryptococcal ligand for NKp30. Studies on the detection of *Candida glabrata* by the related receptor NKp46 suggest that fungal adhesins could be potential ligands for this class of receptors [225].

### 5.6. Eosinophils

Eosinophils are granulocytes that are best known for their roles in allergic responses and parasitic infections (reviewed in [226]). Eosinophilia has been associated with cryptococcal disease in humans and mice [11,227,228,229,230,231,232,233,234,235] and positively correlated to murine susceptibility to cryptococcosis [11,52], but it is not clear if eosinophils have an essential role in the innate immune response to *C. neoformans* or if their recruitment is the byproduct of an ineffectual Th2 response. After infection with *C. neoformans*, eosinophil-deficient ΔdblGATA mice have enhanced Th1 and Th17 responses and decreased lung recruitment of other inflammatory cells, although fungal burden in the lung and brain are not significantly different from WT mice [236]. It is interesting to note that in rats, eosinophils can phagocytose *C. neoformans* and prime T and B cells in order to generate Th1 responses that are protective for the host [233,237,238]. Therefore, the role of eosinophils during cryptococcosis may depend on the particular host setting.

### 5.7. Other Innate Immune Cells

Innate lymphoid cells (ILCs), other than NK cells, have not been extensively studied in cryptococcosis, but type 2 ILCs may be detrimental to host anti-cryptococcal responses [239]. Derivatives of B-1 cells may have direct antifungal effects against *C. neoformans* [102], as discussed earlier in this review. Epithelial and endothelial cells not only serve as a physical barrier to microbial invasion, but can also participate as effector innate immune cells (reviewed in [240,241]). Lung epithelial cells can bind *C. neoformans* and produce cytokines in response to the fungus [242,243], and endothelial cells may enhance anti-cryptococcal activity of neutrophils [244]. The potential role of γδ T cells is still unclear. Mice deficient in γδ T cells have improved infectious outcomes after *C. neoformans* challenge [245], but studies in a protective model of cryptococcosis suggest that γδ T cells are a source of beneficial IL-17A in the setting of neutropenia [208].

## 6. Conclusions

By methodically investigating common mammalian antifungal mechanisms, researchers have established important roles for cellular PRRs, in particular MR, TLR9, and NKp30, and for signal transduction through CARD9 and MyD88 in protective immune responses against *C. neoformans*. Other promising PRR candidates include NLRs like NLRP3 and certain scavenger receptors. Furthermore, soluble mediators including natural IgM and complement have key functions in facilitating host recognition and immunity to *C. neoformans*. Many additional signaling pathways have been studied, but they either require further evaluation as to their specific anti-cryptococcal functions or appear to have limited or even detrimental roles in host responses to *C. neoformans*. Whether the limited findings are due to redundancies in the immune system remains to be determined [246]. Several innate immune cell types appear to have effector functions that facilitate *C. neoformans* clearance and prime adaptive immune responses under certain conditions, but the mechanisms that coordinate these processes require further definition. Much of the work on anti-cryptococcal immunity has been performed in vitro, so it will be important to confirm these pathways in vivo and in human hosts, when possible.

Since *C. neoformans* is equipped with unique virulence factors, like its polysaccharide capsule, that enable it to evade or subvert the host immune response [1], it is not unexpected that the fungus would stimulate distinct innate immune responses compared to other fungal pathogens. Thus, while it is important to study the potential roles of established antifungal pathways in the response to cryptococcosis, it is also critical to work towards identifying immune mechanisms that may be specific to *C. neoformans*. Identification of additional patient populations susceptible to cryptococcosis, such as those with anti-granulocyte macrophage colony-stimulating factor (GM-CSF) autoantibodies [247,248], may reveal previously unknown immune processes important for the host response to *C. neoformans*. Additionally, the rise of new bioinformatics approaches like next-generation sequencing [249] and tools like CRISPR-Cas gene editing [250] and fluorescent probes [251] may enable the discovery of novel pathways in anti-cryptococcal immunity.
